# The Impact of DSM-IV Mental Disorders on Adherence to Combination Antiretroviral Therapy Among Adult Persons Living with HIV/AIDS: A Systematic Review

**DOI:** 10.1007/s10461-012-0212-3

**Published:** 2012-05-30

**Authors:** Sandra A. Springer, Azem Dushaj, Marwan M. Azar

**Affiliations:** Yale AIDS Program, Yale University School of Medicine, 135 College Street, Suite 323, New Haven, CT 06511 USA

**Keywords:** HIV/AIDS, Mental illness, Adherence, Antiretroviral therapy, Systematic review, Persistence, cART, Depression, Anxiety, Psychotic disorders

## Abstract

This is a systematic review of eighty-two published studies investigating the impact of DSM-IV mental disorders on combination antiretroviral therapy (cART) adherence and persistence among persons living with HIV/AIDS (PLWHA). Sixty-two articles examined depression, with 58 % (*N* = 32/62) finding lower cART adherence and persistence. Seventeen articles examined one or more anxiety disorders, with the majority finding no association with cART adherence or persistence. Eighty percent of the studies that evaluated the impact of psychotic (*N* = 3), bipolar (*N* = 5) and personality disorders (*N* = 2) on cART adherence and persistence also found no association. Seven out of the nine studies (78 %) evaluating the impact of antidepressant treatment (ADT) on cART adherence found improvement. Adherence and depression measurements varied significantly in studies; common research measurements would improve data harmonization. More research specifically addressing the impact of other mental disorders besides depression on cART adherence and RCTs evaluating ADT on cART adherence are also needed.

**Electronic supplementary material:**

The online version of this article (doi:10.1007/s10461-012-0212-3) contains supplementary material, which is available to authorized users.

## Introduction

Combination antiretroviral therapy (cART) has greatly improved the morbidity, and decreased the mortality associated with HIV infection [1–3]. The benefits of cART, however, are typically contingent upon excellent cART adherence and persistence in order to achieve suppression of HIV-1 RNA levels and an increase in CD4 T cell lymphocytes [4, 5]. Suboptimal adherence to cART is strongly related to viral proliferation [6, 7], drug resistance [7, 8], disease progression [9], and death [3]. Factors that can impair adherence to cART include drug addiction [10–12], alcohol use disorders [11, 13], low socioeconomic status [14], social stigma [15–17], neurocognitive disorders [18], and mental disorders [19]. When combined, mental disorders and substance use disorders among persons living with HIV/AIDS (PLWHA) synergistically increase mortality via impairing adherence to cART [20].

Mental disorders include a variety of psychiatric conditions and are defined by the fourth Diagnostic Manual of Mental Disorders (DSM-IV) [21] as ‘a clinically significant behavioral or psychological impairment of an individual’s normal cognitive, emotional, or behavioral functioning associated with present distress and caused by physiological or psychosocial factors’. Mental disorders are more common among PLWHA (63 %) as compared to the HIV-negative population (30.5 %) [19, 22]. In a recently published study, HIV-positive men were more likely to have any mood disorder [odds ratio (OR) = 6.10], major depressive disorder/dysthymia (OR = 3.77), any anxiety disorder (OR = 4.02), and any personality disorder (OR = 2.50) when compared to their HIV-negative same-sex counterparts [23]. In another study an estimated 60 % of PLWHA receiving care in North Carolina had co-morbid mental disorder symptoms [24].

The mental disorder most commonly associated with HIV infection is major depressive disorder (MDD) [25] with a prevalence ranging from 16.2 % [26] to 36 % [25]. This is a four- to seven-fold greater prevalence than in the general population (4.9 %) [27]. The large variation in prevalence rates of mental disorders has been partially attributed to differences in the specificity and sensitivity of the study instruments used [28]. Mental disorders have been associated with decreased adherence to cART and impaired HIV virologic control in several studies [14, 29–36]. In one longitudinal study, HIV-positive mothers with co-morbid mental disorders were approximately six times more likely to die than adherent participants with no depressive symptoms [32]. A recently published meta-analysis of 95 independent studies of PLWHA with depressive disorders concluded that depression was significantly associated with nonadherence to cART (*r* = 0.19; 95 % CI = 0.14–0.25, *p* < 0.0001) [37]. This meta-analysis, however, was not a systematic review of the studies of depression and adherence to cART. To the authors’ knowledge there has not yet been a systematic review of studies published in the English literature evaluating all major Axis I and II DSM-IV mental disorders on cART adherence and persistence among adult PLWHA.

The specific aim of this paper is to therefore systematically review studies evaluating the impact of all of major Axis I and II DSM-IV mental disorders, excluding substance use disorders, on combination antiretroviral adherence and persistence among PLWHA. Though major depression is the principal focus of this review due to its higher prevalence, other mental disorders such as anxiety and psychotic disorders will also be examined as they have also been reported to negatively impact participant adherence [34, 38].

## Methods

### Data Search

PubMed, Scopus and Web of Knowledge were queried for peer-reviewed original human research papers published in English from 1996 to December 2011. Google Scholar was also reviewed for details, full text, and additional articles. The search for this systematic review took place from November 2010 to December 2011. Keywords and their combinations used in the search are available in an on-line appendix. Papers were included if they were conducted in other countries outside of the United States, given the high prevalence of mental disorders among PLWHA throughout the world.

### Study Selection and Inclusion/Exclusion Criteria

**Fig. 1:**
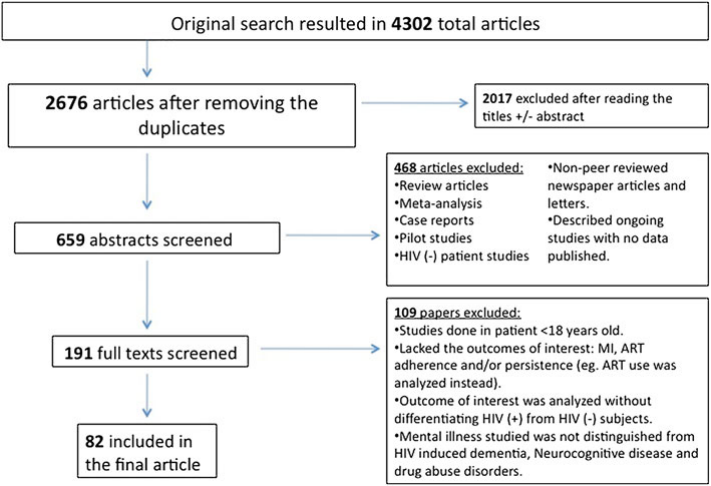
Systematic review study selection flow diagram

Figure 1 represents a PRISMA (Preferred Reporting Items for Systematic reviews and Meta-Analyses) flow diagram for this systematic review [39]. The original search resulted in 4,302 total documents (PubMed—2,151 articles, Scopus—1,847 articles, Web of Knowledge—304 articles) of which 2,676 articles remained after eliminating duplicate articles. Of these, 659 articles met the following inclusion criteria for the primary selection: [1] mental disorders, including psychotic, mood, anxiety, somatoform, and personality disorders as defined by International Classification of Diseases (ICD-9 and ICD-10) [40, 41], Diagnostic and Statistical Manual of Mental Disorders (DSM-IV Axis I or II diagnosis) [21] or mental health symptoms (many studies used screening tools for mental disorder symptoms, rather than specific mental disorder diagnostic measures) [42]); and [2] antiretroviral treatment adherence or persistence. Articles included in this systematic review evaluated the impact of DSM-IV mental disorders or mental health symptoms on at least one of the following cART medication terms: (a) Adherence (synonym: compliance), defined as “the extent to which a participant acts in accordance with the prescribed interval, and dose of a dosing regimen” or “percentage of correctly timed doses (doses taken/doses prescribed × 100)” [43] and (b) persistence, defined as “the duration of time of initiation to discontinuation of therapy (by the participant)” [43].

One reviewer (AD) identified the papers included in the tables provided within this manuscript and in the electronic appendix. A second independent reviewer (MA) applied selection criteria to a random sample consisting of 20 % of articles from the primary selection. Agreement between reviewers was assessed using Cohen’s kappa (κ) statistic and was equal to 0.69 [44]. Where there were differences between the two reviewers, a third reviewer (SAS) made the final decision as to whether the article should be included or excluded from the systematic review. Subsequently, 82 studies met the final inclusion criteria for this systematic review.

### Data Extraction

Standardized data collection forms were used to extract all data including: study authors; study site; year and duration of study; study design; population characteristics; sample size; mental disorder studied and the tool used to screen and diagnose the mental disorder; definition of and the tool used to measure cART adherence. In cases where no standardized tool was used for the mental disorder or cART adherence, the tools used in the studies were noted in the data collection form. The data collection form was similar to Table 1 and the data was extracted at least twice from each article by one single author (AD). The study characteristics in the tables were evaluated by one author (AD). If inconsistencies in definitions of adherence or mental disorder were identified then further appraisal of the particular issue would be carried out by two authors (AD and MZ) and noted in the tables. For example, if it was identified that the study did not use a standardized tool to create a definition of depression, e.g. ‘depression defined as ever being seen by a psychiatrist or taking ADT’, then the problem with the particular definition would be written in the extraction table, e.g. ‘definition of depression was misleading because it may have included persons with a past medical history of depression who are no longer depressed introducing bias into results’.

**Table 1:** Impact of depressive disorders on cART adherence: study characteristics

Author, publication year, location	Study design and evaluation period	PLWHA population, sample size	Adherence: measurement (M), definition (D) and time period (T)	Depressive disorder and scale used to measure depressive disorder	Impact of depressive disorder on adherence
*I. Depressive disorders associated with decreased adherence to cART*					
Arnsten, J. H., X. Li, et al. (2007) USA	Cross-sectional study (2001–2005)	636 adults reporting recent IDU and sexual activity with an opposite-gender partner	M: Self-report D: >90 % adherence T : Previous 1 day	Depression-7-item depression component of the BSI	Multivariate analysis (MVA): Depression was significantly associated with poorer adherence, (OR = 0.74, 95 % CI = 0.58–0.94, *p* < 0.05)
Boarts, J. M., E. M. Sledjeski, et al. (2006) USA	Prospective cohort study (3-month follow-up)	57 participants	M: Self-report (AACTG adapted questionnaire) D: Continuous variable T: Previous week	Depression—CES-D	MVA: Depressive symptoms at baseline predicted lower levels of adherence at follow-up (*R* ^2^ = 0.076, *p* ≤ 0.05)
Buathong, N., N. Hiransuthikula, et al. (2009) Thailand	Cross-sectional study (Oct 2007–Jan 2008)	379 participants on cART recruited from the immunology and sexual transmitted disease clinics	M: Self-report D: ≥95 % adherence T: Previous 1 month	Depression—BDI–II	MVA: Depression was significantly associated with non-adherence (AOR = 4.68; 95 % CI = 2.77–7.88, *p* < 0.001)
Carrieri, M. P., M. A. Chesney, et al. (2003) France	Cohort study (Oct 1995; followed up for the first 18 months on cART)	96 IDU participants (initially adherent to cART)	M: Self-report D: Adherence failure: <80 % adherence or did not “totally” follow their prescribed regimen T: Previous week or at any visit before the 18th month of treatment	Depression—CES–D	MVA: Depression was significantly related to adherence failure (OR = 2.5, 95 % CI = 1.0–6.0, *p* = 0.044)
Carrieri, M. P., C. Leport, et al. (2006) France	Cohort study (May 1997–Jun 1999; 5-year follow-up)	970 participants	M: Self-report (ACTG questionnaire) D: High (100 % adherence), Moderate (80–99.9 % adherence) and Poor (<80 % adherence) T: Previous 4 days	Depression—CES-D (score >16)	MVA: Depression was independently associated with moderate or poor adherence but not with high adherence (Coeff = 0.18, 95 % CI = 0.07–0.29)
Cruess, D. G., S. C. Kalichman, et al. (2011) USA	Prospective cohort study (Mar 2005–Oct 2008)	324 participants	M: Unannounced pill count D: Continuous variable (percentage adherence) T: Previous 3 months	Depression—CES-D	MVA: Higher mean depression score was significantly associated with lower antiretroviral medication adherence (β (4,300) = −0.22, *p* < 0.001)
Diiorio, C., F. McCarty, et al. (2009) USA	Cross-sectional study	236 participants	M: UCSF Adherence Questionnaire D: Reasons for missing medication T: Previous 30 days	Depression—CES-D	MVA: Significant negative association was found between depression and poor adherence (fewer depressive symptoms supported greater adherence)
Do, N. T., K. Phiri, et al. (2010) Botswana (Afrika)	Cross-sectional study (Apr–May 2005)	300 participants	M: Self-report, institutional adherence (pharmacy refill rate), and a culturally modified Morisky scale D: [1] 100 % adherence in the past 4 days, [2] 100 % adherence in the past 1 month, and [3] 100 % refill in the past 3 months T: Previous 4 days, 1 month and prior consecutive 3 months	Depression—BDI	MVA: Depression was a significant predictor of non-adherence (*p* < 0.02)
Etienne, M., M. Hossain, et al. (2010) Kenya, Uganda, Zambia, Nigeria, Rwanda	Cross-sectional study (Aug 2004–Apr 2005)	921 participants	M: Self-report D: Non-adherence: [1] Missing 1 or more doses; [2] missing 1 or more appointments T: Previous week (for doses); previous 3 months (for appointments)	Depression—Factor scores (low, medium, high) constructed from three questions	MVA: Only high depression score was associated with non-adherence (OR = 0.57, 95 % CI = 0.39–0.84, *p* < 0.01)
Gonzalez, J. S., C. Psaros, et al. (2011) USA	Cohort study	91 participants in methadone maintenance	M: EDMs D: 100 % adherence (no missed doses) T: Previous 2 weeks	Depression: clinician ratings (MINI, Clinical Global Impression Scale and MADRS) and self-report (BDI-SF)	MVA: Each unit increase in the Clinical Global Impression Scale was associated with 75 % increased odds of non-adherence (OR = 1.75, *p* = 0.002, 95 % CI = 1.23–2.48). For each SD MADRS increase, there was a 2.6-fold increased odds of non-adherence (OR = 2.60, *p* = 0.001, 95 % CI = 1.45–4.67)
Kacanek, D., D. L. Jacobson, et al. (2010) USA	Longitudinal cohort study (Feb 1995–Dec/2004)	225 participants	M: Self-report D: Suboptimal adherence: missing >5 % of cART doses T: Previous 7 days	Incident depression- Burnam’s interviewer administered 8-item screening tool	MVA: Subjects who developed depressive symptoms had a twofold greater risk of suboptimal adherence at follow up (RR = 1.8, 95 % CI = 1.1–3.0)
Kleeberger, C. A., J. Buechner, et al. (2004) USA	Longitudinal cohort study (Oct 1998–Oct 2000)	597 men	M: Self-report D: Dichotomized: 100 % adherence or less than 100 % adherence T: Previous 4 days	Depression—CES-D	MVA: CES-D score >16 was an independent predictor of decreasing adherence (OR = 1.8, *p* = 0.03)
Lazo, M., S. J. Gange, et al. (2007) USA	Longitudinal cohort study (1999–2004)	1,904 participants (640 men and 1,304 women)	M: Self-report (AACTG questionnaire) D: Dichotomized: 100 % adherence or less than 100 % adherence T : Previous 4 days (men) or 3 days (women)	Symptoms of depression were measured using the CES-D	MVA: Symptoms of depression were a significant independent predictor of decreased adherence among men (OR = 1.44, 95 % CI = 1.06–1.95, *p* < 0.05), but not women
Li, L., S. J. Lee, et al. (2010) Thailand	Cross-sectional study 2007	386 participants	M: Self-report (of failing to adhere to cART) D: <100 % adherence T: Previous 1 month	Depressive symptoms: 15-item screening test developed and used previously in Thailand (Thai Department of Mental Health, 2006)	MVA: Depression was a significant predictor of poor adherence to cART (OR = 0.69, *p* = 0.03)
Olisah, V. O., O. Baiyewu, et al. (2010) Zaria	Cross-sectional study (Sep–Dec 2006)	310 participants	M: Self-report D: Poor adherence: <100 % adherence T: Previous 7 days	Depression—CES-D and SCAN to confirm depression diagnosis	Bivariate analysis (BVA): cART adherence in participants with depressive disorder (36.4 %) was significantly poorer than that in non-depressed participants (78.9 %), (χ^2^ = 34.657, df = 1, *p* < 0.05)
Phillips, K. D., L. Moneyham, et al. (2005) USA	Cross-sectional study	173 low-income, women living with HIV/AIDS in the rural southeastern United States	M: Self-report (AACTG questionnaire) D: Continuous variable T: Previous 1 and 3 months	Depression—CES-D, classified as mild depressive symptoms (CES-D score 8–15) and severe depressive symptoms (CES-D score 16 or greater)	BVA: Severe depressive symptoms were significantly associated with non-adherence (*p* = 0.0002)
Protopopescu, C., F. Raffi, et al. (2009) France	10 year Cohort study (enrolled May 1997–June 1999)	1,010 participants	M: Self-report (AACTG questionnaire) D: Non-adherence: <100 % adherence T: Previous 4 weeks	Depression—CES-D, participants were classified as having depressive symptoms if their CES-D score was >17 for men and >23 for women	MVA: Depressive symptoms were independently associated with non-adherence (Coeff = 0.171, 95 % CI = 0.087–0.283, *p* < 0.001)
Rao, D., B. J. Feldman, et al. (2011) USA	Cross-sectional study (Feb–Nov 2009)	720 participants	M: Self-report (AACTG questionnaire) and VAS D: VAS adherence item was converted into 4-category ordinal scale, perfect adherence = 100 % adherence on VAS T: Previous 4 days and 4 weeks	Depressive symptoms: PHQ-9	MVA: Depressive symptoms had a moderate negative effect on HIV medication adherence (stand. *b* = −0.17, *p* < 0.01)
Rodkjaer, L., T. Laursen, et al. (2010) Denmark	Cross-sectional study (May 2005–Sep 2005)	205 participants	M: Self-report (AACTG questionnaire) D: Non-adherence: <100 % adherence T: Previous 4 days	Depression—BDI-II	MVA: Participants at risk of depression (BDI > 20) were more likely to be non-adherent (OR = 5.7, 95 % CI = 1.7–18.6)
Rodkjaer, L., T. Laursen, et al. (2011) Denmark	Longitudinal cohort study (May 2005–Sep 2008)	205 participants at baseline (in 2005) and 148 participants at follow-up (in 2008)	M: Self-report (AACTG questionnaire) D: Non-adherence: <100 % adherence (in 2005) or stopping cART for 7 days or more during the last 12 months (in 2008) T: Previous 4 days	Depression—BDI-II	MVA: Participants at risk of moderate to major depression (BDI > 20) were more likely to be non-adherent to cART in the 4 days prior to assessment in 2005, and more likely to have stopped cART for 7 days or more during the last 12 months in 2008
Royal, S. W., D. P. Kidder, et al. (2009) USA	Cross-sectional study (July 2004–May 2005)	644 homeless or unstably housed PLWHA	M: Self-report D: Continuous variable T: Previous 2 and 7 days	Depression—CES-D	MVA: Depression was associated with decreased 2 day (AOR = 1.73, 95 % CI = 1.28–2.34, *p* = 0.0003) and 7 days (AOR = 1.91, 95 % CI = 1.35–2.72, *p* = 0.0003) adherence
Sarna, A., S. Pujari, et al. (2008) India	Cross-sectional study (May–Aug 2004)	310 participants	M: Self-report (AACTG questionnaire) D: ≥90 % adherence T: Previous 4 days	Depression-BDI II	MVA: Severe depression (AOR = 4.48, 95 % CI = 1.64–12.27, *p* = 0.003) was associated with lower adherence
Schuman, P. (2001) USA	Cross-sectional study (Dec 1996–Dec 1997)	371 women with advanced HIV disease	M: Self-reported cART adherence D:Self-report of taking >75 % of their cART (as frequently as prescribed or almost all the time) T: Previous 2 weeks	Depression—CES-D (CES-D >15)	MVA: Depressive mood was significantly associated with poorer adherence (OR = 0.34, 95 % CI = 0.18–0.64, *p* < 0.001)
Singh, N., C. Squier, et al. (1996) USA	Longitudinal observational study	46 VA Medical Center participants	M: Computerized pharmacy refill records D: Non-compliant: filling <80 % of medication T: Previous 1 month	Depression—BDI and POMS	MVA: POMS > 42 was significantly associated with non-compliance (OR = 1.4, 95 % CI = 1.1–1.8, *p* < 0.01) but BDI score was not
Spire, B., S. Duran, et al. (2002) France	Prospective cohort study with (May–Oct 1997; follow-up visit after 4 months)	445 participants who were started on PI	M: Self-report D: 100 % adherence T: Previous 4 days	Depression—CES-D	BVA: Baseline depression was not associated with future non-adherence; but an increase in CES-D score during the 4-months period of treatment was significantly associated with non-adherence
Tadios, Y. and G. Davey (2006) Ethiopia	Cross-sectional study (Dec 2004–Jan 2005)	431 participants on cART	M: Self-report D: ≥95 % adherence T: Previous 1, 3 and 7 days	Depression—BDI (cut-off = 14) (depression used as dependent variable)	MVA: Adherence to cART was significantly associated with not being depressed (AOR = 2.8, 95 % CI = 1.5–5.4, *p* = 0.002)
Tucker, J. S., M. A. Burnam, et al. (2003) USA	Cohort study (Jan 1996–Jan 1998)	1910 participants	M: Self-report to 3 adherence questions D: 100 % adherence T: Previous week	Major depression—CIDI-SF of the WHO	MVA: Participants with depression were more likely to be non-adherent than those without a MI. (OR = 1.7, 95 % CI = 1.3–2.3)
Vranceanu, A. M., S. A. Safren, et al. (2008)	Randomized cohort crossover trial (Nov 2002-Jan 2005; total of six study visits, enrollment and five follow-up visits)	156 participants Group 1: Two physician visits with depression screening then crossover to Group 2 Group 2: Two physician visits without depression screening then crossover to Group 1	M: EDMs adherence D: Continuous variable (percent adherence) T: Previous 30 days (which represents the 30-day period after depression measurement)	Depression- Brief (self-report) screening measures of depression (PC-SAD)	MVA: Continuous Depression score was significantly associated with decreased percent adherence (*p* = 0.015). Dichotomous depression score was not
Waldrop-Valverde, D. and E. Valverde (2005) USA	Cross-sectional study	58 IDU participants	M: Self-report D: 100 % adherence T: Previous day	Depression- BDI	MVA: Depression was significantly related to decreasing adherence (OR = 0.924, 95 % CI = 0.863–0.989, *p* = 0.023)
Wagner, G. J., K. Goggin, et al. (2011) USA	Cross-sectional and longitudinal analyses of 10 merged studies (1997–2009)	1,374 participants	M: EDMs (in all studies) D: Continuous variable and dichotomous variable (good adherence: >90 %) T: Previous 2 weeks	Depression—BDI(×1), BDI-II (×4), BSI(×2) and CES-D(×3)	MVA: In cross-sectional multivariate analyses, continuous depression, cognitive depressive symptoms, and severe depression were associated with lower cART adherence. In longitudinal analysis, reductions in both continuous and categorical depression predicted increased cART adherence (b(SE) = −0.015, *p* < 0.01)
*II. Depressive disorder not significantly associated with adherence to cART*					
Berger-Greenstein, J. A., C. A. Cuevas, et al. (2007) USA	Cross-sectional study	85 participants diagnosed with drug abuse & psychiatric disease	M: Self-report (ACTG-adapted questionnaire) D: Continuous variable T: Previous 3 days	MDD–SCID & BDI-II	BVA: MDD was not significantly associated with non-adherence to cART
Bottonari, K. A., S. A. Safren, et al. (2010) USA	Prospective cohort study (3-month follow-up)	87 participants	M: Self-report (AACTG questionnaire) D: Continuous variable; subsequent analyses utilized Box-Cox transformed variables to improve the normality of adherence T: Previous 30 days (1 month)	Depression—MINI and MADRAS	MVA: Neither depression nor depressive severity predicted HIV treatment adherence when controlling for the impact of acute life events
Campos, L. N., M. D. Guimaraes, et al. (2010) Brazil	Prospective cohort study (May 2001–May 2002)	293 participants	M: Self-report D: ≥ 95 % adherence T: Previous 3 days	Depression—HADS	BVA: Depression was not independently associated with non-adherence
Catz, S. L., J. A. Kelly, et al. (2000) USA	Cross-sectional study (Dec 1997 –Aug 1998)	72 participants	M: Self-report (7-point scale) D: Non-adherence: <100 % adherence T: Previous 5 days and 3 months	Depression—CES-D	MVA: No significant association was found between non-adherence and depression
Gibbie, T., M. Hay, et al. (2007) Australia	Longitudinal cohort study (recruited from Jan 2003 to Mar 2004; followed-up for 24 months)	72 participants	M: Self-report D: ≥95 % adherence T: Previous 24 h, 4 days and 7 days	Depression- BDI (cut-off = 10), and SCID	MVA: SCID diagnosis of current major depression was not significantly related to adherence (*b* = −0.99, 95 % CI = 0.052–2.64, *p* = 0.322). BDI was not included in the analysis
Gonzalez, J. S., F. J. Penedo, et al. (2004)USA	Cross-sectional study	90 HIV(+) MSM and women (of any sexual orientation)	M: Self-report (ACTG questionnaire) D: Non-adherence: <100 adherence T: Previous 4 days	Depression—BDI	MVA: BDI score was not significantly related to medication adherence
Gordillo, V., J. del Amo, et al. (1999) Spain	Cross-sectional study (Dec 1997 –May 1998)	366 participants	M: Self-report and pill count method (Returning those pills that had not been taken in the previous month to the pharmacist) D: >90 % adherence T: Previous 1 week and 1 month	Depression—BDI, (cut-off = 14)	MVA: Depression was not an independent predictor of non-adherence
Ingersoll, K. (2004) USA	Cross –sectional study	120 participants	M1: Electronic medical record and self-report D: Four dichotomous non-adherent behaviors: [1] running out of medications, [2] not always taking medications as directed, [3] ≤95 % adherence or [4] having notations of non-compliance in the medical record T: Previous week	Major Depression -CIDI-SF	MVA: Major Depression was not an independent predictor of all four non-adherent behaviors
Johnson, M. O., S. E. Dilworth, et al. (2011) USA	Cross-sectional study (Jan 2009–Sep 2010)	295 HIV-positive men (data were collected from 210 male couples or 420 men)	M: Self-report (AACTG questionnaire and the visual analog (VAS) scale) D: 100 % versus <100 % adherence T: Previous 3 and 30 days	Depression—CES-D	MVA: Depressive symptoms were not associated with suboptimal 3-day or 30-day adherence
Kalichman, S. C., J. Pellowski, et al.(2011) USA	Longitudinal cohort study (Jan 2008–Jun 2009)	179 participants	M: Unannounced pill counts D: Non-adherent: taking <85 % of medications T: For 8 consecutive months	Depression—CES-D	MVA: Depression was not associated with non- adherence
Keuroghlian, A. S., C. S. Kamen, et al. 2011) USA	Cross-sectional study	38 participants	M: Self-report (AACTG questionnaire) D: Adherent: no missed doses T: Previous 4 days	Depression—CES-D	MVA: Depression was not significantly associated with cART adherence (*p* > 0.05)
Kyser, M., K. Buchacz, et al. (2011) USA	Cross-sectional analysis of a prospective cohort study (The SUN study, Mar 2004 - Jun 2006	528 participants	M: Self-report D: Non-adherence: missing at least one dose of medications T: Previous 3 days	Depression—PHQ-9	BVA: Depression was not an independent predictor of non-adherence
Leserman, J., G. Ironson, et al. (2008) USA	Cross-sectional study (Feb 2004–Feb 2007)	105 participants	M: Self-report (AACTG questionnaire) D: Non-adherence: missing at least one dose of medications T: Previous 2 weeks	Depression—BDI	MVA: Depressive symptoms were not associated with non-adherence
Mellins, C. A., J. F. Havens, et al. (2009) USA	Cross-sectional study	542 participants	M: Self-report (AACTG questionnaire) D: 100 % adherence T: Previous 3 days	Major Depression -SCID	BVA: The presence or absence of major depression on the SCID was not associated with adherence
Mohammed, H., L. Kieltyka, et al. (2004) USA	Cross-sectional study (Mar 1999–Aug 2001)	273 participants	M: Self-report D: Non-adherence: <100 adherence T: Previous week	Depression—the following questions: “In the last 7 days did you feel: [1] that you could not shake the blues? [2] depressed? [3] fearful? and [4] that your sleep had been restless?”	MVA: Depression was not found to be significantly associated with non-adherence
Moore, D. J., C. Posada, et al. (2011) USA	Cross-sectional analysis of a cohort study	77 participants	M: EDMs D: Adherent: >90 % adherence T: Previous 30 days	MDD–BDI	BVA: Current or past diagnosis of MDD was not associated non-adherence (*p* > 0.05)
Moss, A. R., J. A. Hahn, et al. (2004) USA	12-month prospective cohort study (Mar 1998 Apr 2001)	148 participants	M: Pill count self-report and EDMs D: [1] Continuous variable [2] cART discontinuation: no pills were taken for 1 month T: Previous 3 days and 1 month	Depressive symptoms—BDI (score >15)	BVA: BDI >15 was not significantly associated with cART discontinuation or with adherence
Mugavero, M., J. Ostermann, et al. (2006) USA	Cross-sectional analysis (Dec 2001–Apr 2002)	474 participants	M: Self-reported D: Non-adherence: <100 % adherence T: Previous 7 days	Depression—BSI	MVA: Depression was not significantly associated with non-adherence (when trauma variable was in the model)
Palmer, N. B., J. Salcedo, et al. (2003) USA	Cross-sectional study	107 participants diagnosed with substance abuse and psychiatric diseases (all on methadone)	M: Self-report (AACTG questionnaire) D: ≥ 95 % adherence T: Previous 3 days	Depression -SCID-I	BVA: Depression was not associated with HIV medication adherence
Shin, S., M. Munoz, et al. (2008) Peru	Cross-sectional study (Nov 2005–Nov 2006)	43 participants with tuberculosis	M: Self-report D: Non-adherence: <100 % adherence T: Previous 4 weeks	Depression—HSCL	MVA: Depression by HSCL (score >1.75) was not significantly associated with non-adherence
Van Servellen, G., B. Chang, et al. (2002) USA	Cross-sectional study	182 participants	M: Self-report and medical records D: Non-adherence: self-report or presence of non-adherence behavior in the medical records T: Previous 3 months	Depression—HADS	MVA: Depression was not shown to be associated with non-adherence
Wagner, G. J., L. M. Bogart, et al. (2011) USA	Cohort study	214 African American males	M: EDMs D: Continuous variable T: Previous 6 months	Depression: 8-item depression scale from the Medical Outcomes Study	BVA: Depression was not associated with adherence
*III. Adherence variable predicts change in depressive symptoms*					
Ammassari, A., A. Antinori, et al. (2004) Italy	Multicenter Cross-sectional study (Nov 1999–Feb 2000)	135 participants^a^	M: Self-report D: Non-adherence: Missing at least 1 dose of cART T: Previous 1 week	Depression—MADRAS (score >19)	MVA: Depression scores were significantly higher in the non-adherent group, compared with the adherent group (*p* = 0.03)
Bianco, J. A., T. G. Heckman, et al. (2011) USA	Cross-sectional study (Jun 2008–Jul 2009)	242 participants (>50 years old adults enrolled in a RCT who had a score of >5 on the GDS)	M: Self-report (ACTG questionnaire) D: Non-adherent: skipping medication at least once and/or taking a medication late at least twice T: Previous 7 days	Depression—Geriatric Depression Scale (GDS)	MVA: Non-adherent participants were significantly more depressed (*b* = −0.05, Wald’s χ^2^ = 4.52, *p* < 0.033)
Catz, S. L., T. G. Heckman, et al. (2001) USA	Cross-sectional study (1997)	84 participants	M: Self-report (six-point Likert scale) D: Dichotomized as ‘consistent adherence’ (No skipped doses) or ‘inconsistent adherence’ (at least 1 skipped dose) T: Previous 7 days	Depression –BDI	BVA: Adherence was not associated with depression
Farley, J., E. Miller, et al. (2010) Nigeria	Cross-sectional study (June-July 2007)	399 participants (222 cART-experienced and 177 cART-naive)	M: Pharmacy refill adherence rate D: Non-adherence: pharmacy refill rate <95 % T: From the time cART was first dispensed until a cutoff date shortly after the study ended	Depression -CES-D Binary cut-off for CES-D scores are defined as ≥16 and ≥21	MVA: Having a pharmacy refill rate <95 %, was associated with a CES-D ≥ 16 (*p* = 0.004) and a CES-D ≥ 21 (*p* < 0.001)
Herrmann, S., E. McKinnon, et al. (2008) Australia	Longitudinal cohort study (Jul 2003-Dec 2005; >6 months follow-up)	357 participants	M: Self-report (AACTG questionnaire) D: 100 % adherence T: Previous month	Depression-CES-D scale (depression used as dependent variable)	BVA: Non-adherent participants scored higher values on the depression indicator scale (*p* = 0.03)
Springer, S. A., S. Chen, et al. (2009) USA	6-month RCT	89 IDU participants	M: Self-report (ACTG questionnaire) D: Continuous variable (reported as a mean change in percentage adherence) T: Previous 3 days	Depression—CES-D, MDD was defined as having a CES-D score >16 (depression used as dependent variable)	MVA: Adherence to cART was associated with improved depressive symptoms. Increased adherence was significantly associated to decreased CES-D score (*p* = 0.01)
*IV. Depressive disorder associated with decreased persistence on cART*					
Carrico, A. W., E. D. Riley, et al. (2011) USA	Cohort study (using data from a RCT)	603 participants	M: Self-report D: cART utilization classified as: [1] continuous cART utilization-being on cART at baseline and remaining on cART; [2] cART discontinuation-stopping cART and remaining off during any subsequent follow-up assessments; and [3] intermittent cART utilization-stopping and restarting cART at least once T: 25 months of follow-up	Depression—BDI-I	MVA: Depression at baseline independently predicted a 39 % increase in the odds of cART discontinuation (OR = 1.39, 95 % CI = 1.08–1.78, *p* < 0.01)
Li, X., J. B. Margolick, et al. (2005) USA	Cohort study (Apr 1999–Mar 2002)	687 MSM	M: Self-report (of cART persistence) D: [1] Continuing cART— not stopping medications for 2 or more consecutive days [2] Interrupted cART—stopping cART for >2 consecutive days at least once; and [3] Discontinued cART—stopping all ART between and still off cART at end of assessment period T: Previous 6 months (time between Vi and Vi+1)	Depression—CES-D	MVA: CES-D score >16 was an independent predictor for Interrupting cART, (OR = 1.97, 95 % CI = 1.38–2.80), and Discontinuing cART, (OR = 2.03, 95 % CI = 1.24–3.32) but not for continuing cART
Maru, D. S., R. D. Bruce, et al. (2008) USA	6-month RCT of 2:1 to DAART versus SAT	141 drug users	M: EDMs, observed doses, and self-report. Both persistence and adherence were measured D: The time-to-drop-out (persistence) was considered to be the duration from the first observed dose of DAART to the last DAART visit the participant received T: Previous 6 months	Depression—CES-D	BVA: The presence of severe score on the CES-D scale predicted time-to-DAART discontinuation (HR = 2.4, 95 % CI = 1.0–6.0, Gehan statistic = 4.4; *p* < 0.05)
*V. Depressive disorder associated with increased persistence on cART*					
Himelhoch, S., C. H. Brown, et al. (2009) USA	Longitudinal cohort study (2000–2005)	4989 participants	M: Self-report D: cART discontinuation: the participant either went off cART but remained active in care or dropped out of active care T: Previous year	Depressive disorder was defined using ICD-9	MVA: The hazard probability for cART discontinuation among those with depressive disorders was significantly lower in the first year (AOR = 0.61, 95 % CI = 0.54–0.69); but it did not significantly differ in subsequent years

*AACTG* Adult AIDS Clinical Trials Group, *ADT* Anti-Depressant Therapy, *AOR* Adjusted Odds Ratio, *BDI (-II)* Beck Depression Index (2nd Edition), *BSI* Brief Symptom Inventory, *BVA* Bivariate Analysis, *CES-D* Center for Epidemiologic Studies-Depression Scale, *CIDI-SF* Composite International Diagnostic Interview-Short Form, *DAART* Directly Administered Antiretroviral Therapy, *GDS* Geriatric Depression Scale, *cART* combination Antiretroviral Therapy, *HADS* Hospital Anxiety and Depression Scale, *HSCL* Hopkins Symptom Checklist-15, *HR* Hazard Ratio, *ICD-9-CM* International Classification of Diseases, 9th Revision, Clinical Modification, *IDU* Injection Drug User, *IES* Impact of Event Scale, *IRR* Incidence Rate Ratio, *MADRAS* Montgomery-Asberg Depression Rating Scale, *EDMs* Electronic Drug Monitors, *MDD* Major Depressive Disorder, *MSM* Men who have sex with men, *MINI* Mini International Neuropsychiatric Interview, *MVA* Multivariate analysis, *OR* Odds Ratio, *PLWHA* People Living with HIV/AIDS, *POMS* Profile of Mood States depression factor scale, *PHQ-9* 9- item Patient Health Questionnaire, *RCT* Randomized Control Trials, *RH* Risk Hazard ratio, *RR* Relative Risk, *SAT* Self-administered therapy, *SCAN* Schedule for Clinical Assessment in Neuropsychiatry, *SCID-IV* Structured Clinical Interview for DSM-IV, *SD* Standard Deviation, *VAS* Visual Analog Scale

^a^Participants: People Living with HIV/AIDS (PLWHA) >18 years old

In order to clearly present the results, the data were separated into four categories according to the mental disorder that was the main focus of the studies. Table 1 summarizes studies that evaluated ‘the impact of depressive disorders on cART adherence and persistence’. These constituted the majority of articles reviewed (62/82; 76 %). Tables 2, 3 and 4 are presented as an electronic appendix. Table 2 summarizes studies that examined the ‘impact of specific mental disorders other than depression on cART adherence and persistence’. Table 3 presents studies that evaluated the ‘impact of mental disorders not specified per DSM-IV diagnostic criteria on cART adherence and persistence’. Finally, Table 4 summarizes articles that evaluated ‘the impact of pharmacologic antidepressant treatment (ADT) on cART adherence’ among PLWHA with comorbid depressive disorders. Due to the length and scope of the manuscript, behavioral treatments of depressive disorders were not included in this systematic review. In cases when cART persistence was measured instead of cART adherence, the term used by the authors was noted in the tables.

## Results

This systematic review examines 82 studies evaluating the impact of mental disorders and mental disorder symptoms (including mood, anxiety, psychotic, and personality disorders) on two primary outcomes: adherence to, and persistence on cART (see Tables 1 in the manuscript and the Tables 2, 3 and 4 within the electronic appendix).

### Antiretroviral Adherence Measurements

Adherence to cART was assessed using multiple methods (as highlighted in the Tables). Seventy-seven of the articles used only one measurement of adherence, four studies used two adherence measurements and one used three different measures of cART adherence. These included: self-reports (participants recall) (*N* = 65), electronic drug monitors (EDMs) (*N* = 9), pharmacy refill record review (*N* = 6), hospital or clinic record review (*N* = 2), unannounced pill counts (*N* = 5), and directly observed therapy (DOT) (*N* = 1), and some of the studies used a combination of the aforementioned measures of adherence to cART (*N* = 5).

The time period over which adherence was assessed ranged from 1 day to 1 year prior to the interview. Among the self-report instruments, adherence recall periods ranged from: 1 day to 4 weeks; 7 days (the most commonly used recall period) (*N* = 22 studies); 4 weeks (*N* = 12 studies); 4 days (*N* = 12 studies); 3 days (*N* = 10 studies); 2 days (*N* = 6 studies); 3 months (*N* = 4 studies); 1 day (*N* = 4 studies); 2 weeks (*N* = 3 studies), and 5 days (*N* = 1 study). Adherence was reported either as a continuous or binary (dichotomous) measure, either in association with mental disorders or as the proportion of the sample meeting a specified level of adherence. There was considerable variation for binary (dichotomous) adherence measure and its cut-off, differentially defined by studies as ≥75 % adherence, ≥80 % adherence, ≥90 % adherence, ≥95 % adherence, 100 % adherence, no missed doses, or a specific number of missed doses for a given period of time.

### Antiretroviral Persistence Measurements

Among five studies that measured cART regimen persistence, four used self-report measures and one used EDMs, self-report, and DOT combined. The time period over which persistence was assessed ranged from 6 months to 3 years.

### Measures of Mental Disorders

In addition to ICD-9, ICD-10 and DSM-IV diagnostic criteria, other validated and non-validated screening tools (see Table 1 in the manuscript and Tables 2 and 3 in the electronic appendix) were used to screen for and diagnose specific mental disorders and evaluate mental disorder symptoms. The most commonly evaluated mental disorder was depressive disorder (*N* = 62), followed by anxiety disorders (*N* = 17), bipolar disorder (*N* = 5), psychotic disorders (*N* = 3), personality disorders (*N* = 2), and adjustment disorder (*N* = 2). Some studies evaluated more than one mental disorder and used more than one instrument to evaluate the presence of one or more mental disorders. Depressive disorder and depressive symptoms were measured by using one or two self-report scales in 60 studies whereas two studies used a diagnosis of depression (ICD-9 code) on participants’ medical charts. The most common instrument employed to assess depression and depressive symptoms was the Center for Epidemiological Studies Depression Scale (CES-D) [45, 46], (*N* = 23; 37 %) followed by the Beck Depression Inventory (BDI and BDI-II) [47–50] (*N* = 18; 29 %) and the Structured Clinical Interview for DSM-IV (SCID) [51] (four studies; 6 %), with the remaining studies using seventeen other scales: Montgomery–Asberg Depression Rating Scale (*N* = 3) [52], Brief Symptom Inventory (BSI) (*N* = 3) [53], Hospital Anxiety and Depression Scale (HADS) (*N* = 2), Composite International Diagnostic Interview-Short Form (CIDI-SF), Mini International Neuropsychiatric Interview (MINI) (*N* = 2), ICD-9 (*N* = 2), Hopkins Symptom Checklist-15, Burnam’s interviewer administered 8-item screening tool, Schedule for Clinical Assessment in Neuropsychiatry (SCAN), Profile of Mood States (POMS) depression factor scale, Brief (self-report) screening measures of depression (PC-SAD) [54], Geriatric Depression Scale (GDS) [55], Patient Health Questionnaire (PHQ-9) (*N* = 2) [56], the 8-item depression scale from the Medical Outcomes Study, the 15-item screening test developed and used previously in Thailand (Thai Department of Mental Health, 2006), and unspecified instruments constructed from 3 to 4 questions (*N* = 2 studies) [57, 58].

### Primary Outcomes Analysis

Two outcomes were evaluated in this systematic review: (a) cART adherence and (b) cART persistence. Adherence and persistence data for each article were extracted and are presented in Tables 1 in the manuscript; and Tables 2, 3, and 4 in the electronic appendix. The following sections are grouped based on each mental disorder and the effect on these two outcomes.

#### Adherence to cART

##### Impact of Depressive Disorders on cART Adherence

Fifty-eight studies evaluated the impact of depressive disorder (as either the dependent or independent variable) on cART adherence. These data are presented in Table 1.

*Depressive Disorders Associated with Decreased Adherence to cART*. Depressive disorders were significantly associated with decreased cART adherence in thirty out of 52 studies (57 %). Among these thirty studies, thirteen were cohort [59–71], one was a randomized control trial (RCT) [72], and 16 were cross-sectional studies [29, 57, 73–86]. The majority of these studies used self-report measures for assessing the cART adherence variable (*N* = 25/30, 83 %), while the other five studies used EDMS, pharmacy refill data and unannounced pill counts to assess adherence. Depression symptom measures were used in the majority of these studies to assess the variable ‘depression’, with the CES-D (*N* = 13) and the BDI (*N* = 7) as the most common measures. Twenty-seven of the thirty studies (90 %) used multivariate analysis to evaluate the association with depressive symptoms/diagnosis and poor cART adherence.

*Depressive Disorders not Significantly Associated with Adherence to cART*. Twenty-two studies did not find a statistically significant association between depression and decreased cART adherence. Sixteen were cross-sectional [34–36, 58, 87–98], whereas six were cohort studies [38, 99–103]. The majority of these studies utilized a diagnosis of depressive disorder at baseline (either via the SCID, MINI, CIDI, HADS, or ICD-9 code) as the depression variable (*N* = 10), while seven studies used a depressive symptom scale, and the remainder of the studies utilized other non-standardized measures to determine depressive symptoms/disorder, such as the HSCL and a non-specified scale. Multivariate analyses were reported for finding an association with depressive disorder or depressive symptoms and cART adherence in the majority of these studies. The majority of studies utilized self-report measures for the adherence variable.

*Adherence Variable Predicts Depressive Symptoms*. In six studies, depression was the dependent variable while adherence to cART was the independent variable [104–109]. Five of these studies reported an association with non-adherence to cART and higher depressive symptoms [104–108]. The one study that did not find a statistically significant association between cART adherence and depression had a relatively smaller sample size (*N* = 84) as compared to the studies that did find an association, was completed earlier than the other studies (year 1997), and only used a six-point likert scale to measure adherence [109].

##### Impact of Anxiety Disorders on cART Adherence

Table 2 lists seventeen articles that evaluated the impact of one or more anxiety disorders on cART adherence. Anxiety disorders included: (i) unspecified anxiety disorder (*N* = 8), (ii) generalized anxiety disorder (GAD, *N* = 3), (iii) posttraumatic stress disorder (PTSD, *N* = 9), (iv) panic disorder (*N* = 3), (v) agoraphobia (*N* = 1), or a combination of the above (see Table 2 in the electronic appendix).

*Impact of PTSD on cART Adherence*. The relationship between PTSD and cART adherence was examined by nine studies (four cohort, and five cross-sectional studies). Two studies (one prospective cohort and one cross-sectional) found that PTSD was significantly associated with decreased cART adherence [33, 34]. Both of these studies utilized self-report adherence measures and examined results with multivariate analyses. One of these studies used the Post-Traumatic Diagnostic Scale (PDS) and the Impact of Event Scale (IES) while the other study used the IES only.

In one study, PTSD participants were significantly more likely to adhere to cART than depressed participants. In this study, 69 participants diagnosed with PTSD or depression were classified into four groups according to the severity of symptoms: (i) control (low PTSD/low depression); *N* = 22; (ii) PTSD (high PTSD/low depression), *N* = 11; (iii) depressed (low PTSD/high depression), *N* = 12); and (iv) mixed (high PTSD/high depression), *N* = 24). The PTSD group was significantly more likely to adhere to cART regimens compared to the depressed group during the previous week (OR = 23.9, 95 % CI = 1.607–356.075) and during the previous 2 weeks of the study (OR = 27.55; 95 % CI = 1.99–381.82) [110].

Six studies (three cross-sectional, two cohort and one RCT) found no significant association between PTSD and cART adherence [36, 59, 72, 94, 103, 111]. Adherence was measured using self-report instruments in five studies and EDM in one study. PTSD was measured using the SCID (*N* = 2), PDS (*N* = 2), IES (*N* = 1) and a short form of the widely used Davidson Trauma Scale (SPAN, *N* = 1).

*Impact of Panic Disorder on cART Adherence*. Three studies examined the effect of panic disorder on cART adherence. A study of 1,910 participants found that participants with panic disorder were more likely to be non-adherent to cART than those who were not diagnosed with a mental disorder after being screened with the full CIDI [68]. In the remaining two cross-sectional studies, panic disorder diagnosed using the SCID-I was not significantly associated with cART adherence [36, 94]. These two studies used self-report instruments to measure adherence to cART.

*Impact of Generalized Anxiety Disorder (GAD) on cART Adherence*. The impact of GAD on cART adherence was assessed in three reviewed articles.

One study reported that participants with GAD were more likely to be non-adherent than those without a mental disorder [68]. This cohort study of persons with a diagnosis of GAD measured adherence by the ACTG 7-day recall method, while the other two articles that found no significant association between GAD and cART adherence were cross-sectional evaluations and used the ACTG 3-day recall method [36, 94]. In one of these studies the participants had to have associated panic disorder in addition to the GAD, therefore making the specific association with GAD difficult to assess.

*Impact of Other Anxiety Disorders (Agoraphobia, Unspecified Anxiety Disorder) on cART Adherence*. Agoraphobia was evaluated as a separate disorder in one cross-sectional study of 542 participants, and was not found to be significantly associated with cART adherence [36]. This study used a self-report instrument to measure adherence to cART and agoraphobia was diagnosed using the SCID.

Eight studies examined the association between an unspecified anxiety disorder (defined as either anxiety symptoms or anxiety disorders in general) and cART adherence. In three articles, anxiety symptoms were significantly associated with decreased cART adherence [10, 38, 111]. In these three studies, adherence was measured using self-report instruments (*N* = 2) and pharmacy refill (*N* = 1). Anxiety symptoms were measured using the Hospital Anxiety and Depression Scale (HADS, *N* = 1), State-Trait Anxiety Inventory (STAI, *N* = 1), and BSI (*N* = 1). One of these studies reported the result based on multivariate analysis and two of them reported the results based on bivariate analysis. Four studies found no statistically significant association between anxiety and non-adherence [60, 83, 96, 109]. In these four studies, adherence was measured using self-report instruments. Anxiety symptoms were measured using Hospital Anxiety and Depression Scale (HADS, *N* = 1), State-Trait Anxiety Inventory (STAI, *N* = 1), Symptom Check List-90-Revised (*N* = 1), and a self-administered questionnaire/face-to-face interview of somatic symptoms of anxiety (*N* = 1). Three of these studies reported the result based on multivariate analysis whereas only one of them reported the results based on bivariate analysis. In another study of unspecified anxiety disorder, anxiety symptoms significantly predicted that participants adhered to cART [91]. In this cross-sectional study of 120 participants with non-specific anxiety symptoms measured with the CIDI-SF scale, cART adherence was measured using four dichotomous non-adherent questions based on electronic medical records.

##### Impact of Psychotic Disorder (Schizophrenia) on cART Adherence

Two cross-sectional studies did not find a statistically significant association between schizophrenia/psychotic disorder and cART adherence [94, 112]. In one of these studies the presence of a psychotic disorder among mentally ill participants was confirmed by a referring mental health professional, while in the other study the presence of psychotic disorder among triply diagnosed participants with HIV, substance abuse and mental disorders was confirmed using the SCID. Both studies used self-report measures to evaluate adherence to cART, however one study assessed this with the ACTG 3-Day recall and the other used self report and EDMs.

##### Impact of Personality Disorders on cART Adherence

Two cross-sectional studies examined the association between personality disorders (borderline and antisocial) and cART adherence. Both of these studies utilized the self-report ACTG 3-day recall adherence measure, but they differed in the subject populations. One study of HIV+ persons enrolled in multi-site cohort study found no statistically significant association between borderline or antisocial personality disorders and adherence [36]. The other study of 107 HIV+ participants with co-occurring substance use disorders enrolled in a methadone program found that borderline personality disorder was associated with non-adherence to cART, while antisocial personality disorder was not [94].

##### Impact of Bipolar Disorder on cART Adherence

The effect of having bipolar disorder on cART adherence was examined in four cross-sectional studies. One study reported that bipolar disorder was significantly associated with decreased adherence to cART [35] and utilized EDMs as the adherence tool. In three studies, bipolar disorder was not significantly associated with decreased adherence to cART [36, 94, 112]. Adherence tools and subject populations differed between these three studies.

##### Impact of Somatization, Dissociation, Adjustment Disorder and Unspecified Mental Disorders on cART Adherence

One study found that levels of somatization uniquely predicted adherence problems in a multivariate model [109]. Another study found that dissociation moderated the effect of PTSD on adherence resulting in lower odds of cART adherence (OR = 0 0.95, *p* < 0.05). PTSD symptoms were significantly associated with lower odds of adherence in individuals reporting high levels of dissociation (OR = 0.86, *p* < 0.05) but not in those reporting low levels of dissociation (OR = 1.02, *p* > 0.05) [34].

Two cross-sectional studies found no significant association between adjustment disorder and cART adherence [36, 94]. Both of these studies utilized the ACTG 3 -day recall for cART adherence assessment.

Six studies included in this systematic review assessed the impact of unspecified mental disorders on adherence to cART. In these papers, mental disorder was either used as a general term or combined with more than one mental disorder into one variable (see Table 3 in the electronic appendix). All six studies (four cross-sectional and two cohort) reported a significant association between the mental disorder and decreased adherence to cART [6, 113–117]. Adherence was measured using self-report instruments in five studies and EDMs in one study. Five different instruments were used to measure the presence of a mental disorder (see Table 3 in the electronic appendix).

#### Persistence on cART

##### Impact of Depressive Disorder on cART Persistence

Three studies, two longitudinal cohort and one RCT, found that depression was associated with decreased cART persistence [31, 118, 119]. Another study found less cART discontinuation in the first year among those with depressive disorder, but there was no association with cART persistence after the first year [120].

##### Impact of Bipolar Disorder on cART Persistence

One cohort study concluded that HIV+ Medicaid beneficiaries with severe affective disorder (defined as having bipolar disorder plus major depressive disorder identified by ICD-9 codes) were significantly less persistent in their use of cART than those without serious mental illness [121].

##### Impact of Psychotic Disorder on cART Persistence

In one cohort study, psychotic disorder (schizophrenia) identified by ICD-9 coding was not significantly associated with persistence on cART [121].

##### Impact of Unspecified Mental Disorder on cART Persistence

One cohort study of 4,989 PLWHA found that compared to participants with no mental disorders, the hazard probability for discontinuing cART was significantly lower in the first and second years of treatment among those with a severe mental disorder (defined as ICD-9 codes for schizophrenia, other psychoses and bipolar disorder). Among participants with mental disorders, those with six or more mental health visits per year were significantly less likely to discontinue cART compared with participants with no mental health visits [120].

#### Impact of Antidepressant Treatment on Adherence to cART

Nine studies (seven cohort studies, one RCT, and one cross-sectional study) evaluated the impact of pharmacologic ADT on adherence to cART among depressed PLWHA (see Table 4 in the electronic appendix). Seven studies reported that pharmacologic treatment for depression was significantly associated with increased cART adherence [69, 122–127], while in one study ADT of depression was associated with decreased cART adherence. In this study, however, pharmacotherapy was NOT specifically evaluated. The authors defined treatment of depression as: ‘seeing a psychiatrist, receiving a diagnosis of depression, or being prescribed ADT’ [11]. Therefore, the construct validity of this study is problematic since the employed definition of psychiatric treatment did not distinguish the treatment of depression from a diagnosis of depression.

A RCT reported that participants receiving directly observed fluoxetine for the treatment of depressive symptoms had similar cART adherence compared to participants in the referral arm [128]. This study, however, had high degrees of continuation of cART at the end of the study in both arms (73 vs 75 %), suggesting an overall high degree of persistence on therapy.

## Discussion

DSM-IV mental disorders, especially depressive disorders, are extremely common among PLWHA and have been associated with decreased adherence to cART and increased mortality [29, 32, 57, 73]. The literature concerning HIV and mental disorders is staggering, yet the data has yet to be rigorously appraised. Although a meta-analysis studying the effect of depression on cART adherence was recently published [37], this is to our knowledge the first systematic review to examine the impact of all mental disorders, with the exception of substance use disorders, on cART adherence and persistence among PLWHA.

The majority of papers included in this review studied the impact of depression on cART adherence and persistence. Thirty of the 52 studies (57 %) that studied adherence as the dependent variable, five out of six studies (83 %) that studied adherence as the independent variable, and three out of the four studies (75 %) that examined persistence reported that a depressive disorder was associated with a decrease in cART adherence and persistence. Among these studies, 90 % based their results on multivariate analysis compared to 60 % of the studies that did not find an association between depression and cART adherence and persistence. One study reported a decreased probability of cART discontinuation among those with depressive disorders in the first year but not in subsequent years [120]. This study, however, did not measure adherence in itself, but rather cART discontinuation (cART persistence) and defined it broadly as ceasing cART or dropping out of active care. The 23 studies that found no significant association between depression and adherence and persistence to cART also tended to have a smaller sample size (mean sample size = 195) as compared to the 35 studies that found positive associations (mean sample size = 477).

Some of the reasons for why the studies of depressive disorders found differing associations with adherence to cART were likely attributed to the differences in whether they utilized specific depression screening measures/symptom rating scales (CES-D, BSI, PHQ) or standard diagnostic tools of depressive disorder (SCID, MINI, CIDI) to define the variable of ‘’depressive disorder”. Most of the studies that did not report a significant association with adherence utilized more standard diagnostic tools of depressive disorder such as the SCID, MINI, and CIDI. It would be important for future research evaluations of depression in HIV adherence research to improve data harmonization by utilizing the categorization of depression instruments that Simoni et al. [42] described as follows: [1] standardized diagnostic interviews, that are commonly used to assess the categorical diagnosis of depression based on DSM or ICD criteria, (including in this category CIDI, MINI, SCID, HADS, BDI etc.) [2] depression screening instruments, that provide empirically based cut-offs and are useful as the basis for referrals to more comprehensive evaluations or to estimate the prevalence of possible depression (including in this category CES-D and HSCL etc.), and [3] symptom-rating scales, that are useful for monitoring change in depression symptoms over time (including in this category BDI, CES-D, HSCL, MADRS etc.) [42]. Although there is no gold-standard for evaluation of depression, it has been recommended that researchers need to make informed choices based on the characteristics of the study population and the purpose of the research [42]. It is clear from this review that such rigorous evaluation of the study intentions needs to be applied in order to improve data harmonization efforts in the future.

Other causes of differences in results of the depression studies were likely due to the heterogeneity of the diagnostic and/or screening tools used for assessing depression. For instance the CES-D, identified as a standardized tool to screen for depressive symptoms, was the most commonly used instrument among the papers included in this review and it has twenty items, but not all versions are DSM-IV compatible (i.e., early versions do not include items that map directly onto DSM symptoms) [42]. Perhaps some of the variability in studies evaluating the effect of depression on cART adherence may have been associated with the different versions of the CES-D as well as the different cut-offs used to assess severity of depressive symptoms. Uniform assessment of depression is essential in order to improve efforts at data harmonization across studies of PLWHA. Harmonization of data can increase the likelihood of finding an association with a common outcome variable such as depression and adherence to cART. Furthermore, if the goal of the research study is to identify individuals with a clinical diagnosis of major depressive disorder, investigators should consider following up individuals who screen positive for depressive symptoms with a full diagnostic interview such as the SCID, MINI or CIDI, all of which have been validated with PLWHA in both developed and developing countries [42, 129]. Investigators should also be careful when using medical records (ICD-9 or DSM-IV codes) to identify participants as having a diagnosis of ‘depression’, as was done in a few of the evaluated studies. This particular method may misidentify a previous diagnosis of depression in a possibly successfully treated participant thereby affecting the analysis of the adherence outcome variable.

The majority of the studies (*N* = 17/20 articles) evaluating the impact of ‘specific mental disorders other than depression’ on cART adherence and persistence (see Table 2 in the electronic appendix) assessed anxiety disorders. Anxiety disorders were associated with decreased adherence in seven of the studies [unspecified anxiety disorder (*N* = 3), GAD (*N* = 1), PTSD (*N* = 2), panic disorder (*N* = 1)]; but no association was detected in the majority of the studies (*N* = 15)) [Unspecified anxiety disorder (*N* = 4), GAD (*N* = 2), PTSD (*N* = 6), panic disorder (*N* = 2), and agoraphobia (*N* = 1). Although two studies [unspecified anxiety disorder (*N* = 1), PTSD (*N* = 1)] reported data suggesting that anxiety disorders were associated with increased adherence to cART, one study was conducted with a control group of participants with high levels of depression, rather than a typical control group without any mental disorder [110]; and the second study, concluded that “screening positive for any anxiety disorder reduced the risk of failing to take medications as directed” [89]. Although the gross sample size of these studies seems sufficient, the number of affected participants for each specific anxiety disorder may in effect not have been large enough to reach statistical significance. Given the high lifetime prevalence of anxiety disorders among PLWHA (7 %) [130], however, studies evaluating individual anxiety disorders are not sufficient in number. To obtain a better understanding of the impact of specific mental disorders on cART adherence other than depression, future well-designed prospective cohort studies and RCTs with larger sample sizes for each specific disorder should be conducted to assess for causality.

All of the studies assessing the impact of ‘unspecified mental disorders’ on cART adherence concluded that mental disorders are associated with decreased adherence to cART. Importantly though, all of these studies included depression/depressive symptoms as the mental disorder variable [6, 113–117]. The result of these studies could therefore be attributed to the direct effect of the depressive disorder on adherence to cART. Only one study, which assessed cART persistence as the dependent variable, concluded that a severe mental disorder was significantly associated with lower probability for cART discontinuation in the first and second years (suggesting increased cART persistence), compared to participants with no mental disorders [120]. An additional finding of this study was that persons with a mental disorder and with six or more mental health visits per year were significantly less likely to discontinue cART compared to those with no mental health visits. It is therefore plausible that increased mental health visits in this study contributed to improved cART persistence by enhancing linkage to health care, including HIV-associated health-care [120].

The treatment of depression with antidepressants was found to be associated with improved adherence in seven of the nine studies (78 %) examining this effect. One of these studies had a very small sample size of only nine participants on ADT, therefore rendering any conclusions questionable [122]. Three of the four studies that found improved adherence to cART among participants adherent to ADT used pharmacy records to measure adherence [123, 125, 127]. The use of pharmacy records as the sole indicator of adherence could confound the adherence outcome as participants who obtain their antidepressant medications directly from pharmacies are more likely to pick up their cART medications, thus “hiding” the direct effect of ADT on cART adherence (via the improvement of depressive symptoms). Furthermore, adherence to one medication may indicate a propensity towards adherence in general. Therefore participants who are adherent to ADT may be more likely to adhere to cART simply because they are more adherent as a rule, and not because of improved depressive symptoms. In one study that did not find an association between ADT and cART adherence, starting psychiatric treatment was broadly defined as receiving a diagnosis of depression, seeing a psychiatrist or taking ADT, therefore making the individual effects of pharmacological, and non-pharmacological psychiatric treatment impossible to unfurl [11]. A recent RCT of 137 HIV+ homeless and marginally housed persons found no significant difference in cART adherence among those receiving directly observed fluoxetine as compared to the those referred to the community for psychiatric care [128]. This study however had high levels of persistence to cART at the end of study in both groups (73 vs 75 % NS), as well as similar percentages of viral suppression in both groups (specific data not reported). These results suggest that perhaps persistence to cART may be more significant than adherence for a durable cART regimen.

Issues regarding differences between the studies in specific ADTs as well as appropriate individual dosing, however, make interpretation of the impact of ADT on cART adherence for depression difficult. Little evidence from randomized controlled clinical trials is available to guide the psychiatric treatment of PLWHA [131]. Some studies, however, have shown that depression can be effectively treated in PLWHA [132–134], specifically using selective serotonin reuptake inhibitors (SSRIs) [135, 136]. Despite some possible evidence based from this review that ADT may improve adherence to cART among PLWHA who have co-morbid depression, it is still unclear at this time whether treating depressive disorders with ADT improves adherence to cART. Although other ADT besides SSRIs including citalopram [137] and tricyclic antidepressants [136, 138], have been shown to be effective in treating depression among PLWHA, SSRIs [136, 139] are better tolerated in PLWHA and recommended as first-line agents for depression [140]. In devising research involving antidepressants for PLWHA, however, clinicians should control for the stage of HIV illness, co-morbid illnesses such as hepatitis B and C, the potential for drug interactions with cART, type of ADT, maximal dosing and participants’ preferences [133, 141–143].

This systematic review also identified a large heterogeneity of measurements and definitions of adherence to cART in various studies likely contributing to the variation in outcomes. Overall the majority of studies utilized some form of self-report, while fewer utilized EDMS, pharmacy refill reviews, and unannounced pill counts. There is no universally accepted ‘gold’ standard for the measurement of cART adherence [144]. Self-report adherence measures and other indirect measures such as EDMs and pill counts have been found to distinguish between clinically meaningful patterns of medication-taking behavior [145, 146]. Self-report, however, tends to overestimate adherence by 10–20 % as compared to EDMs evaluations because self-report is susceptible to recall bias, inaccurate memory and potentially to social desirability bias [147–149]. Pharmacy record review, on the other hand, has been show to misclassify participants as non-adherent to cART in up to 43 % of the time when disregarding leftover medication [150]. The variations in measurements utilized to evaluate adherence to cART in the reviewed studies, as well as the definition of recall period, likely contributed to the great variation of the primary outcome, cART adherence.

There are some limitations to this review. Although we were very rigorous with inclusion criteria and exclusion criteria, it is plausible that some relevant papers that should have been included were missed. Additionally, the exclusion of behavioral treatments for depression and other mental disorders constitutes a limitation when discussing the scope of mental disorder treatments. Behavioral treatments have overall been found to be effective in the treatment of mental disorders, but due to the length of the paper and the large quantity of data to be processed in the scope of pharmacological treatment alone, it was decided to not include studies on behavioral treatments in this particular review. The inclusion of studies from both developed and developing countries also could have introduced bias into our findings since confounding factors such as barriers and facilitators of cART adherence may be specific to different geographic areas [151–153]. In developing countries, food insufficiency [154–156] and health system deficiencies have been found to undermine treatment continuity and adherence to cART [157], whereas religiosity has been shown to be positively correlated with cART adherence [158]. Conversely, examining data from different parts of the world increases the generalizability of our findings and emphasizes the widespread nature of both mental disorders and HIV.

Despite the limitations of the review, this is the first paper to exhaustively review studies evaluating the association between DSM-IV mental disorders (excluding substance use disorders) and adherence and persistence to cART among adult PLWHA. Most of the studies included in this review studied the adherence variable at a time period when cART was more complex, requiring multi-dose regimens. Current preferred once-daily dosing regimens, however, have been recently shown to improve adherence [159–162]. In a time when cART complexity has decreased while potency of regimens has increased, persistence to cART may be a superior indicator of HIV outcomes than cART adherence and a better variable to evaluate [4, 159, 163, 164]. Recent studies have found data suggesting that adequate cART adherence may not be the key factor in viral suppression [165]. Future studies should evaluate the impact of mental disorders and their treatment on HIV outcomes other than adherence such as maximal viral suppression (i.e. HIV VL <48 copies/mL), neurocognitive deficits, as well as liver and renal dysfunction.

## Conclusions

The majority of the studies included in this systematic review identified a significant association between depressive symptoms/disorder and cART nonadherence and non-persistence. Data related to the impact of specific mental disorders other than depression (anxiety disorders, bipolar disorder, psychotic disorders and personality disorders) on adherence to cART are insufficient and inconsistent. Future research should focus specifically on each of these mental disorders and cART adherence. Additionally, common measures should be used to assess cART adherence as well as specific mental disorders to improve data harmonization across studies. Furthermore, the majority of existing data suggest that treatment of depression with ADT may be associated with improved adherence to cART among PLWHA. Future RCT-designed studies will need to be conducted to best determine the association between improvement of depressive symptomatology and cART adherence. Lastly, HIV outcomes other than adherence to cART, such as maximal HIV viral load suppression and neurocognitive deficits are important to evaluate now that more potent combination antiretroviral regimens are available.

## Electronic supplementary material

Below is the link to the electronic supplementary material.

Supplementary material 1 (DOC 118 kb)
