# Supplementary material for: The Impact of DSM-IV Mental Disorders on Adherence to Combination Antiretroviral Therapy Among Adult Persons Living with HIV/AIDS: A Systematic Review
Source: AIDS Behav. 2012 May 30;16(8):2119–43. doi: 10.1007/s10461-012-0212-3 (PMC3481055; doi:10.1007/s10461-012-0212-3)
Supplement: Supplementary file 1 — Supplementary material 1 (DOC 118 kb) [file 10461_2012_212_MOESM1_ESM.doc]

**Electronic Supplementary Materials**

Title: *The Impact of DSM-IV Mental Disorders on Adherence to Combination Antiretroviral Therapy among Adult Persons Living with HIV/AIDS: A Systematic Review*

Authors: *Sandra A..Springer, Azem Dushaj, Marwan Azar*

*This material supplements but does not replace the content of the peer-reviewed paper published in AIDS and Behavior.

**Electronic Appendix:**

**SEARCH TERMS**

*1. To assess studies involving adherence, we used the following search terms:*

adherence, compliance, persistence

*2. To assess studies involving HIV, we used the following search terms:*

acquired immunodeficiency syndrome, AIDS, antiretroviral therapy, antiretroviral treatment, ART, HAART, highly active antiretroviral therapy, HIV, HIV treatment, human immunodeficiency virus

*3. To assess studies involving mental illness, we used the following search terms:*

acute anxiety disorder

affective disorder

antisocial personality

anxiety

avoidant personality

bipolar disorder

body dysmorphic disorder

borderline personality

dependent personality

depression

depressive disorder

dysthymia

GAD

generalized anxiety disorder

histrionic personality

hypochondriac

hypochondriasis

manic disorder

mental health

mental illness

mood disorder

mood disorder

narcissistic personality

panic disorder

personality disorder

personality disorders

post traumatic anxiety disorder

posttraumatic anxiety disorder

post-traumatic anxiety disorder

psychiatric disease

psychiatric disorder

psychiatric illness

psychosis

psychotic disorder

PTSD

schizophrenia

somatization

somatoform disorders

**Table 2: Impact of Specific Mental Illnesses Other Than Depression on c**ART Adherence: Study Characteristics

| Author, Publication Year, Location | Study Design and Evaluation Period | PLWHA Population, Sample Size | Adherence: Measurement (M), Definition (D) and Time period (T) | Mental Illness (MI) & Scale (S) Used to Measure Mental Illness | Impact of Mental Illness on Adherence | | | |
| --- | --- | --- | --- | --- | --- | --- | --- | --- |
|  |  |  |  |  | Anxiety Disorders | Bipolar Disorder | Psychotic Disorders | Personality Disorders |
| Boarts, J. M., E. M. Sledjeski, et al. (2006) USA | Prospective cohort study (3-month follow-up) | 57 participants* | M: Self-report (AACTG adapted self-report) interview  D: Continuous variable  T: Previous week | MI:PTSD  S: Post-traumatic Diagnostic Scale | MVA: PTSD symptoms predicted lower adherence at follow-up only when baseline levels of adherence were removed from the analyses (p≤ 0.05). | X | X | X |
| Boarts, J. M., B. A. Buckley-Fischer, et al. (2009) USA | Prospective cohort study (3-4 month follow-up) | 84 participants divided into 3 groups (59 completed study):  1-No PTSD, *n* =38  2-HIV-related PTSD, *n=*13  3-Non-HIV-related PTSD, *n* =23 | M: Self-report (AACTG adapted self-report) interview  D: Continuous variable  T: Previous week | MI:PTSD  S: Post-traumatic Diagnostic Scale and IES | MVA: The non-HIV-related PTSD group reported significantly lower adherence compared to the non-PTSD group (p< 0.05). In the HIV-related PTSD group, adherence increased over time; while the in non-HIV-related PTSD group adherence decreased over time. | X | X | X |
| Campos, L. N., M. D. Guimaraes, et al. (2010) Brazil | Prospective cohort study (May 2001-  May 2002) | 293 participants | M: Self-report.  D: Non-adherence: <95% adherence  T : Previous 3 days | MI: Anxiety  S: HADS | BVA: Severe anxiety was independently associated with non-adherence (RH= 1.87, 95% CI= 1.14–3.06; p < 0.05). | X | X | X |
| Carrieri, M. P., M. A. Chesney, et al. (2003) France | Cohort study (Oct 1995, followed -up for the first 18 months on cART) | 96 initially adherent IDUs participants | M: Self-report.  D: Adherence failure: <80% adherence or did not “totally” follow their prescribed regimen | MI: Anxiety  S: self-administered questionnaire and the face-to-face interview of somatic symptoms of anxiety | MVA: Anxiety was not significantly related to adherence failure. | X | X | X |
| Catz, S. L., T. G. Heckman, et al. (2001) USA | Cross-sectional study (1997) | 84 participants | M: Self-report (six-point Likert scale)  D: Dichotomized “consistent adherence”: No skipped dose; or “inconsistent adherence”: at least 1 skipped dose.  T: Previous 7 days | MI1: Anxiety  MI2: Somatization  S: Symptom Check List-90-Revised | BVA: Adherence was associated with lower levels of somatization (OR = 0.45, p < 0.05), but not with anxiety. | X | X | X |
| Escobar, I., M. Campo, et al. (2003) Spain | Cross-sectional study (Nov 2000 - Jan 2001) | 283 participants on cART for at least 6 months | M: Pharmacy refill  D: Non-adherence: <95% adherence  T: Previous 4–6 months. | MI: Anxiety  S: State-Trait Anxiety questionnaire (STAI) | BVA: Non-adherence risk increased 3.49-fold (95% CI=2.02-6.02) in those who scored >75% on the anxiety scales. | X | X | X |
| Ingersoll, K. (2004) USA | Cross –sectional study | 120 participants | M1: Electronic medical record and self-report.  D: Four dichotomous non-adherent behaviors: (1) running out of medications, (2) not always taking medications as directed, (3) ≤ 95% adherence or (4) having notations of non-compliance in the medical record.  T: Previous week. | MI: Anxiety Disorders  S: CIDI-SF | MVA: In a stepwise logistic regression anxiety significantly predicted adherence. | X | X | X |
| Keuroghlian, A. S., C. S. Kamen, et al. (2011) USA | Cross-sectional study | 38 participants | M: Self-report (AACTG questionnaire)  D: Adherent: no missed doses  T: Previous 4 days | MI1: PTSD  MI2: Dissociative experience  S1: IES-R  S2: DES-II | MVA: PTSD was associated with lower odds of cART adherence  (OR = 0.92,  p < 0.05). | X | X | MVA: PTSD symptoms were significantly associated with lower odds of adherence in individuals reporting high levels of dissociation (OR = .86, p < .05) but not in those reporting low levels of dissociation (OR = 1.02, p > .05).Dissociation moderated the effect of PTSD on adherence, resulting in lower odds of adherence (OR =0 .95, p < 0.05). |
| Mellins, C. A., J. F. Havens, et al. (2009) USA | Cross-sectional study (Data from a multisite cohort study of 1138 HIV infected adults) | 542 participants | M: Self-report (AACTG questionnaire)  D: 100% adherence  T: Previous 3 days | MI1: Bipolar Disorder  MI2: GAD  MI3: Panic Disorder  MI4: Agoraphobia  MI5: PTSD and  MI6: Adjustment Ds  MI7: Borderline PD  MI8: Antisocial PD.  S: SCID | MVA: GAD, Panic Disorder, Agoraphobia, PTSD and Adjustment Disorder were not associated with adherence. | MVA: Bipolar Disorder was not associated with adherence. | x | MVA: Borderline PD and Antisocial PD were not associated with adherence. |
| Moore, D. J., C. Posada, et al. (2011)  USA | Cross-sectional analysis of a cohort study. | 77 participants | M: EDMs  D: Adherent: >90% adherence  T: Previous 30 days | MI1: Bipolar disorder (type I & II)  S1: SCID  S2: YMRS (manic symptoms)  S3: BDI (depressive symptoms) | X | MVA: Bipolar Disorder participants were significantly less likely to be adherent to cART (P<0.001). | X | X |
| fNilsson Schönnesson, L., M. L. Williams, et al. (2007) Sweden | Cross-sectional study (Nov 2000-Apr 2001) | 193 participants | M: Self-report (AACTG-modified questionnaire)  D: Suboptimal Adherence: (1) to dose instructions taking <95% of the prescribed pills and (2) <100% adherence to scheduled instructions.  T: Previous 4 days | MI1: PTSD-  S1: Impact of Event Scale  MI2: Anxiety  S2: BSI | MVA: Suboptimal adherence to dose instructions was associated with anxiety symptoms (OR = 5.507, 95% CI = 1.787–16.968).  PTSD was not significantly associated with suboptimal adherence to dose instructions. | X | X | X |
| Palmer, N. B., J. Salcedo, et al. (2003) USA | Cross-sectional study | 107 diagnosed with HIV/AIDS, substance abuse and psychiatric diseases (all on methadone) | M: Self-report (AACTG questionnaire)  D: ≥ 95% adherence  T: Previous 3 days | MI1: Bipolar I and II,  MI2: Panic disorder  +/- Agoraphobia, PTSD, GAD, and adjustment disorders  MI3: Nonmood psychotic disorder, mood disorder with psychotic features.  MI4: Borderline PD  MI5: Antisocial PD  S: SCID | BVA: Panic disorder  With Agoraphobia, Panic disorder without Agoraphobia, PTSD, GAD, and adjustment disorders were not associated with adherence. | BVA: Bipolar I and II diagnoses were not associated with adherence. | BVA: Nonmood Psychotic disorder and mood disorder with psychotic features were not associated adherence. | BVA: BPD was significantly associated with non-adherence to HIV meds (*p<*0.05). But antisocial PD was not. |
| Sledjeski, E. M., D. L. Delahanty, et al. (2005) USA | Cross-sectional comparative study | 69 participants divided into 4 groups:  1-Control (low PTSD/low depression); *n* =22  2-PTSD (high PTSD/low depression), *n=*11  3-Depressed (low PTSD/high depression), *n* =12),  4-Mixed (high PTSD/high depression), *n=24* | M: Self-report (AACTG-adapted questionnaire)  D: Dichotomized: 100% adherence or less than 100% adherence  T: Previous 2 days, 1 week and 2 weeks | MI: PTSD  S: IES | MVA: PTSD group was significantly more likely to be adherent during the past week (OR=23.9, 95% CI= 1.607–356.075) and during the past 2 weeks (OR=27.55; 95% CI=1.99–381.82) compared to the depressed (reference) group. | X | X | X |
| Tucker, J. S., M. A. Burnam, et al. (2003) USA | Cohort study (Jan 1996-Jan 1998) | 1910 participants | M: Self-report to 3 adherence questions  D: 100% adherence  T: Previous week | MI1:GAD,  MI2: Panic disorder  S:Short-Form (Followed by full version) of the WHO CIDI-SF. | MVA: Patients with GAD (OR=2.4; 95% C= 1.2-5.0), or panic disorder (OR=2.0; 95% CI= 1.4 -3.0) were more likely to be non-adherent than those without a MI. | X | X | X |
| Van Servellen, G., B. Chang, et al. (2002) USA | Cross-sectional study | 182 participants | M: Self-report and medical records.  D: Non-adherence: presence of non-adherence behavior in the medical records or self-report  T: Previous 3 months | MI: Anxiety  S: HADS | MVA: Anxiety was not associated with adherence. | X | X | X |
| Vranceanu, A. M., S. A. Safren, et al. (2008) | Randomized cohort crossover trial (Nov 2002-Jan 2005) | 156 participants  Group 1: Two physician visits with PTSD screening then crossover to Group 2  Group 2: Two physician visits without PTSD screening then crossover to Group 1 | M: EDMs adherence  D: Continuous variable (percent adherence)  T: Previous 30 days | MI: PTSD:  S: SPAN, a brief self-report screening measure (a short form of the widely used Davidson Trauma Scale) | MVA: Continuous PTSD score was not associated with percent adherence either alone or in a model that included depression. | X | X | X |
| Wagner, G. J., D. E. Kanouse, et al. (2003) USA | Cross-sectional 2-week study | 47 mentally ill participants | M: Self-report and EDMs  D: Continuous variable (expressed as a percentage and mean rates of % adherence)  T: Previous 3 days and 2 weeks | MI1:Bipolar depression,  MI2:Schizophrenia,  MI3:Schizoaffective disorder  MI4: Major depression with psychotic features.  S: Confirmed by the referring mental health professional | X | BVA: Mean rates of adherence varied widely by psychiatric diagnosis but were not statistically significant. | BVA: Mean rates of adherence varied widely by psychiatric diagnosis but were not statistically significant. | X |
| Wagner, G. J., L. M. Bogart, et al. (2011) USA | Cohort study | 214 African  American males | M: EDMs  D: Continuous variable  T: Previous 6 months. | MI1: PTSD  S: PDS | BVA: Combination ART adherence did not differ between those who met criteria for a PTSD diagnosis (M = 58%, SD = 31%) and those who did not (M = 61%, SD =28%) | X | X | X |
| Waldrop-Valverde, D. and E. Valverde (2005) USA | Cross-sectional study | 58 IDUs | M: Self-report.  D: 100 % adherence  T: Previous day. | MI: Anxiety  S:State-Trait Anxiety Inventory | MVA: Anxiety was not significantly related to adherence. | X | X | X |
| Walkup, J. T., U. Sambamoorthi, et al. (2004) USA | Cohort study (Jan 1996-Dec 1998) | 2459 Medicaid beneficiaries | M: Self-report  D: cART persistence: binary variable indicating use of PIs/NNRTIs for each quarter after initiating therapy.  T: 6-36 months. | MI1: Schizophrenia  MI2: Severe affective disorder (Bipolar affective disorder and major depressive disorder, recurrent episode)  S: ICD-9-CM | X | MVA: Patients with severe affective disorder were significantly less persistent in their use of PI/NNRTI therapy than those without serious mental illness (OR = 0.73, 95% CI = 0.57- 0.94, p<0.01). | MVA: Schizophrenia was not associated with persistence of PI/NNRTI therapy. | X |

**Participants:* *People Living with HIV/AIDS (PLWHA) >18 years old*

AACTG: Adult AIDS Clinical Trials Group

(A)OR: (Adjusted) Odds Ratio

ART: Antiretroviral Therapy

BSI: Brief Symptom Inventory

BVA: Bivariate analysis

CIDI-SF: Composite International Diagnostic Interview

DES-II: The Dissociative Experiences Scale-II

EDMs: Electronic Drug Monitors

GAD: Generalized Anxiety Disorder

cART: combination Antiretroviral Therapy

HADS: Hospital Anxiety and Depression Scale

ICD-9-CM: International Classification of Diseases, Ninth Revision, Clinical Modification

IDUs: Injection drug users

IES: Impact of Event Scale

MVA: Multivariate analysis

NNRTIs: Non–Nucleoside Reverse Transcriptase Inhibitors

NRTIs: Nucleoside Reverse Transcriptase Inhibitors

PD: Personality Disorder

PDS/PTDS: Post-traumatic Stress Diagnostic Scale

PI: Protease Inhibitor

PLWHA: People Living with HIV/AIDS

PTSD: Post-Traumatic Stress Disorder

SCID-IV: Structured Clinical Interview for DSM-IV

YMRS: Young Mania Rating Scale

**Table 3: Impact of Unspecified Mental Illness on cART Adherence/Persistence**: Study Characteristics

| **Author, Publication year, Location** | **Study Design and Evaluation Period** | **PLWHA Population, Sample Size** | **Adherence: measurement (M), definition (D) and time period (T)** | **Mental illness (MI) Studied and Scale (S)** | **Impact of MI on Adherence** |
| --- | --- | --- | --- | --- | --- |
| 1. **Impact of Unspecified Mental Illness on cART Adherence** | | | | | |
| Adewuya, A. O., M. O. Afolabi, et al. (2010) Nigeria | Cross-sectional study | 182 participants* | M: Self-report (Morisky Medication Adherence Questionnaire)  D: Ordinal variable as “low” “medium” and “high” adherence  T: Previous week | MI: Psychopathology  S: General Health  Questionnaire (GHQ–12) | BVA: Presence of psychopathology was significantly associated with low adherence (OR = 4.36, 95% CI = 1.83–10.43, B = 3.33 p<0.001) |
| Grierson, J., R. L. Koelmeyer, et al. (2011) Australia | Cross-sectional study (Oct 2008 - Apr 2009) | 867 participants | M: Self-report  D: Non-adherence: difficulty taking cART  T: Previous 2 days | MI: Lifetime diagnosis of a mental disorder | MVA: Lifetime diagnosis of a mental disorder was significantly associated with difficulty taking cART (p<0.05). |
| Kumar, V. and W. Encinosa (2010) USA | Cross-sectional study, (Aug 1997-Jan 1998) | 1,192 participants | M: Self-report to 4 questions  D: Ordinal variable: 1 = did not miss any, 2 = missed dose/s for only 1 day, 3 = missed dose/s for two or 3 days, and 4 = missed dose/s for four or more days  T: Previous week | MI: Depressive and/or anxiety symptoms  S: Two “Mental health problems scales” were constructed based on symptom frequency | MVA: Patients with high depressive/anxiety symptoms had lower odds of adherence than those with low symptoms at mean medication complexity (OR= 0.78; p≤ 0.05). This association, however, varied by cART medication complexity. |
| Soto Blanco, J. M., I. Ruiz Pérez, et al. (2005) Spain | Cross-sectional study (2002) | 281 inmates | M: Self-report (SMAQ questionnaire)  D: Non-adherence: >2 doses missed in the past week, or > 2 days of total non-medication in the last 3 months  T: Previous 1 week, 3 months | MI: Anxiety and Depression  S: Structured questionnaire with interviewer (unspecified) | MVA: Non-adherence was significantly associated with suffering anxiety or depression in the previous week (OR= 2.07, 95% CI= 1.18–3.66, p= 0.01). |
| Mellins, C. A., E. Kang, et al. (2003) USA [_ENREF_41](#_ENREF_41) | Cohort Study (1998-1999) | 97 mothers | M: Self-report (AACTG questionnaire)  D: Continuous variable (percentage adherence)  T: Previous 2 days | MI: Depression; Anxiety disorders (panic, PTSD etc); and psychosis  S: Clinical Diagnostic Questionnaire- presence or absence of any psychiatric disorder in the past 30 days | BVA: Presence of psychiatric diagnosis at baseline was significantly associated to the percentage of missed pills (in past 2 days) at Follow-up 2. (OR=14.30, 95% CI= 2.88–71.00, p< 0.01). |
| Paterson, D. L., S. Swindells, et al. (2000) USA | Prospective cohort study (Aug 1997 -Mar 1999) | 81 participants on PI | M: EDMs  D: >95% adherence  T: Previous 6 months | MI: Schizophrenia, depression, or bipolar affective disorder.  S: BDI, the General Health Questionnaire and medical chart review. | MVA: Active psychiatric illness was an independent risk factor for adherence < 95% (P=0.04). Lower psychiatric morbidity was significantly associated with adherence >95%, (OR=1.7, 95% CI=1.0 -3.0, *p*= 0.04). |
| **II. Impact of Unspecified Mental Illness on cART persistence** | | | | | |
| Himelhoch, S., C. H. Brown, et al. (2009) USA | Longitudinal cohort study (2000–2005) | 4989 participants | M: Self-report  D: cART discontinuation  T: Previous year | MI: Severe MI was defined as having schizophrenia, other psychoses or bipolar disorder.  S: ICD-9. | MVA: Relative to those with no psychiatric disorders, the hazard probability for cART discontinuation was significantly lower in the first and second years among those with a SMI (first year AOR= 0.57, 95% CI= 0.47-0.69; second year AOR=0.68, 95% CI=0.52-0.89). Among those with psychiatric diagnoses, those with six or more mental health visits in a year were significantly less likely to discontinue cART compared with patients with no mental health visits. |

**Participants: People Living with HIV/AIDS (PLWHA) >18 years old*

AACTG: Adult AIDS Clinical Trials Group

(A)OR: (Adjusted) Odds Ratio

BDI: Beck Depression Index

BVA: Bivariate analysis

cART: combination Antiretroviral Therapy

ICD-9-CM: International Classification of Diseases, Ninth Revision, Clinical Modification

EDMs: Electronic Drug Monitors

MVA: Multivariate analysis

PLWHA: People Living with HIV/AIDS

PTSD: Post-Traumatic Stress Disorder

Table 4: Impact of Treatment of Depression on Adherence to cART Among Depressed HIV+ Persons: Study characteristics

| Author, Publication year, Location | Study Design and Evaluation Period | HIV(+) Population, Sample Size | Adherence: Measurement (M), Definition (D) and Time period (T) | Depressive disorder (MI) Scale (S) and ADT treatment (Tx) | Impact of Antidepressant Treatment (ADT) on Adherence |
| --- | --- | --- | --- | --- | --- |
| Akincigil, A., I. B. Wilson, et al. (2011) USA | Retrospective, observational study (secondary data analysis, Jan 2003 - Mar 2007) | 1150 participants* enrolled in a plan with prescription drug and mental health benefits for at least 12 months. | M: Pharmacy record review  D: Adherence: MPR >90%  T: For the period between cART initiation and  discontinuation (cART episode) | MI: MDD and MDD recurrent disorder  S: ICD-9 codes: 296.2-MDD single episode, 296.3-MDD recurrent episode, 311, 300.4, 293.83, 296.90, 309.1, 296.99.  Tx: Antidepressants and/or psychotherapy use | MVA: Depression treatment (antidepressants and/or psychotherapy use) significantly increased the likelihood to be adherent to cART (AOR = 2.52, 95% CI 1.40, 4.53). Antidepressant MPR of >80% were significantly more likely to be adherent to cART than those with poor antidepressant  adherence (AOR = 2.68, 95% CI 1.82, 3.94).  Psychotherapy was not significantly associated with better cART adherence. |
| Cruess, D. G., S. C. Kalichman, et al. (2011) USA | Prospective cohort study (Mar 2005 - Oct 2008) | 324 participants | M: Unannounced pill count  D: Continuous variable (percentage adherence)  T: Previous 3 months | MI: Depression  S: CES-D  Tx: psychotropic medications | MVA: Greater adherence to psychotropic medications regardless of medication class was positively related to higher  cART adherence (β (1, 101)=0.26, p=0.009). |
| Dalessandro, M., C. M. Conti, et al. (2007) Italy | Prospective cohort study | 17 depressed participants (9 patients on ADT treatment) | M: Self-report.  D: Non-adherence: <100% adherence  T: Previous 2 days | MI: Depression  S: MADRS  Tx: Fluvoxamine,  Sertraline or  Paroxetine. | BVA: The comparison of the pre- and post- ADT questionnaires showed a significant improvement in the level of adherence (p< 0.0001). |
| Glass, T. R., M. Battegay, et al. (2010) Switzerland | Prospective Cohort study (Jan 2003-Jan 2009, 4.5 year-follow-up) | 6709 participants | M: Self-report (Simplified SHCS adherence questionnaire)  D: Ordinal variable: Number of missed doses (daily, more than once a week, once a week, once every second week, once a month, never)  T: Previous 4 weeks | MI: Psychiatric illness  S: Having a psychiatric illness as defined by medical records?  Tx: Psychiatric treatment was defined as seeing a psychiatrist, diagnosis of depression, or taking ADT. | MVA: Starting psychiatric treatment was significantly associated with worsening adherence (OR= 1.26, 95% CI= 1.04 -1.52). |
| Horberg, M. A., M. J. Silverberg, et al. (2008) USA | Retrospective cohort study (Jan 2000 - Dec 2003) | 3359 participants | M: Pharmacy database records  D: >90% adherence  T: Previous 12 months | MI: Depression  S: Coded outpatient or inpatient depression diagnosis based on clinical evaluation of the patient.  Tx: SSRIs | MVA: Depression without SSRI use was associated with significantly decreased odds of achieving >90% adherence to cART (OR= 0.81, 95% CI= 0.70 - 0.98, p= 0.03). Depressed patients compliant with SSRIs had cART adherence statistically similar to non-depressed patients taking cART. |
| Kumar, V. and W. Encinosa (2009) USA | Cross-sectional study (Aug 1997- Jan 1998) | 1,192 participants on cART at the second HCSUS follow-up interview. | M: Self-report to 4 questions  D: Ordinal variable:  1 = did not miss any,  2 = missed dose/s for only 1 day,  3 = missed dose/s for two or 3 days  4 = missed dose/s for four or more days.  T: Previous week | MI: Depressive and/or anxiety symptoms  S: Two “Mental health problems scales” constructed based on symptom frequency.  Tx: Antidepressant medications | MVA: Untreated higher depressive and/or anxiety symptoms were strongly associated with non-adherence to cART (OR = 0.72, p< 0.05) but their adherence improved with the use of ADT as the cART complexity increased. |
| Tsai, A. C., S. D. Weiser, et al. (2010) USA | Community-based prospective cohort study with assessments conducted every 3 months (Apr 2002-Aug 2007) | 158 homeless and marginally housed persons with CD4+ T-cell<350/μL and BDI- II score >13 | M: (1) probability of being on an cART regimen; (2) self-reported cART adherence, and (3) probability of reporting complete (100%) cART adherence  D: Continuous variable (percentage adherence)  T: Previous 7 days | MI: Depression  S: BDI- II score>13  Tx: Antidepressant medications (84.3% SSRIs) | MVA: ADT use increased the probability of antiretroviral uptake (weighted OR= 3.87, 95% CI= 1.98-7.58, *p*< 0.001). Self-reported adherence to cART increased by 25 percentage points (95% CI= 14 -36; *p*< 0.001), and the odds of reporting complete adherence nearly doubled (weighted OR= 1.94, 95% CI= 1.20-3.13, *p*= 0.006). |
| Tsai, A. C., D. R. Bangsberg, et al. (2012 (?)) USA** | Non-blinded, Randomized controlled trial (DOT Fluoxetine vs referral to the community for psychiatric care, Jul 2002 - Feb 2008) | 137 HIV+ homeless and marginally housed persons | M: Unannounced pill count  D: Continuous variable (percent cART adherence)  T: Previous 7 days | MI: Depression symptoms  S: Ham-D and BDI-II  Tx: Fluoxetine (DOT) | BVA: Participants receiving DOT fluoxetine had similar average percent cART adherence compared to participants in the referral arm (b=0.05; 95% CI, -0.02 to 0.12; p=0.20). |
| Yun, L. W., M. Maravi, et al. (2005) USA | Retrospective cohort study (Jan 1997-Dec 2001) | 1713 participants (375 patients on ADT) | M: Pharmacy records.  D: >95% adherence.  T: Six months was the minimal observation period | MI: Depression –  S: ICD-9 diagnosis present in administrative data, chart review or electronic pharmacy records  Tx: Antidepressant medications | MVA: cART adherence was lower among depressed patients NOT on ADT (vs. on ADT) (p= 0.012). Adherence to cART was higher among patients adherent to ADT (vs. nonadherent to ADT) (P=0.0014). |

**Participants: People Living with HIV/AIDS (PLWHA) >18 years old*

***The article is in press not published yet.*

ADT: Anti-Depressant Treatment

(A)OR: (Adjusted) Odds Ratio

BDI (-II): Beck Depression Inventory (2nd Edition)

BVA: Bivariate analysis

DOT: Directly Observed Treatment

cART: combination Antiretroviral Therapy

Ham-D: Hamilton Depression Rating Scale

ICD-9-CM: International Classification of Diseases, Ninth Revision, Clinical Modification

MPR: Medication Possession Ratio

MVA: Multivariate analysis

PLWHA: People Living with HIV/AIDS

SSRIs: Selective Serotonin Reuptake Inhibitors
